# 59182 An exploratory analysis of network bridges in translational research; a case study of research grants collaboration networks at University of Rochester School of Medicine and Dentistry

**DOI:** 10.1017/cts.2021.682

**Published:** 2021-03-30

**Authors:** Reza Yousefi Nooraie, Elizabeth Wayman, Ann Dozier

**Affiliations:** University of Rochester Medical Center

## Abstract

ABSTRACT IMPACT: This analysis helps disentangle various paths to translational collaboration, with implications for departmental capacity building and support. OBJECTIVES/GOALS: Studies that bridge research collaboration networks are cross-disciplinary and translational. We explored the characteristics of researchers and their collaboration patterns in bridging research grants at University of Rochester School of Medicine and Dentistry. METHODS/STUDY POPULATION: the database of sponsored research grants from 2011 to 2018, obtained from an internal University database was transformed into a two-mode network of grant-to-investigator. Grants at 90th percentile and above of normalized two-mode betweenness centrality were defined as ‘bridging grants’. For each grant we extracted the gender, academic rank, academic degree, affiliating department, and centrality-status (being at 75th percentile of degree centrality in one-mode collaboration network) of the Principal Investigator (PI), as well as the number of co-investigators (CI) and the existence of central actor(s) in the research team. RESULTS/ANTICIPATED RESULTS: Out of 2491 sponsored grants, 250 were ‘bridging grants’. The significant predictors of bridging were centrality of PI, existence of central CI(s), PI holding PhD, and larger number of CIs. The PI’s academic rank (being full professor) and gender were not significant predictors. Among bridging grants 79 included both central PI and CIs (central actors group) and 60 included no central actor on the team. In the latter group, more PIs were clinical faculty and fewer were full professors. Network analysis of affiliating departments showed that Medicine was the prominent actor in the central actors group, while the network of no-central actor group was more fragmented with Neurology as central. DISCUSSION/SIGNIFICANCE OF FINDINGS: Widely recognized researchers are more likely to collaborate with each other in bridging studies possibly marginalizing less experienced peers. Bridging grants led by less central researchers, often clinician-scientists, may thrive where supportive culture and departmental facilities exist.

